# SPTBN1 Prevents Primary Osteoporosis by Modulating Osteoblasts Proliferation and Differentiation and Blood Vessels Formation in Bone

**DOI:** 10.3389/fcell.2021.653724

**Published:** 2021-03-19

**Authors:** Xuejuan Xu, Jiayi Yang, Yanshi Ye, Guoqiang Chen, Yinhua Zhang, Hangtian Wu, Yuqian Song, Meichen Feng, Xiaoting Feng, Xingying Chen, Xiao Wang, Xu Lin, Xiaochun Bai, Jie Shen

**Affiliations:** ^1^Department of Endocrinology, The First People’s Hospital of Foshan, Foshan, China; ^2^Department of Endocrinology, The Third Affiliated Hospital of Southern Medical University, Guangzhou, China; ^3^The Third Subcommittee on Clinical Medicine, Southern Medical University, Guangzhou, China; ^4^Department of Orthopaedics and Traumatology, Nanfang Hospital of Southern Medical University, Guangzhou, China; ^5^Shunde Hospital of Southern Medical University (The First People’s Hospital of Shunde), Foshan, China

**Keywords:** osteoporosis, osteoblast, SPTBN1, VEGF, STAT1/Smad3 pathway 3

## Abstract

Osteoporosis is a common systemic skeletal disorder that leads to increased bone fragility and increased risk of fracture. Although βII-Spectrin (SPTBN1) has been reported to be involved in the development of various human cancers, the function and underlying molecular mechanisms of SPTBN1 in primary osteoporosis remain unclear. In this study, we first established a primary osteoporosis mouse model of senile osteoporosis and postmenopausal osteoporosis. The results showed that the expression of SPTBN1 was significantly downregulated in primary osteoporosis mice model compared with the control group. Furthermore, silencing of SPTBN1 led to a decrease in bone density, a small number of trabecular bones, wider gap, decreased blood volume fraction and number of blood vessels, as well as downregulation of runt-related transcription factor 2 (Runx2), Osterix (Osx), Osteocalcin (Ocn), and vascular endothelial growth factor (VEGF) in primary osteoporosis mice model compared with the control group. Besides, the silencing of SPTBN1 inhibited the growth and induced apoptosis of mouse pre-osteoblast MC3T3-E1 cells compared with the negative control group. Moreover, the silencing of SPTBN1 significantly increased the expression of TGF-β, Cxcl9, and the phosphorylation level STAT1 and Smad3 in MC3T3-E1 cells compared with the control group. As expected, overexpression of SPTBN1 reversed the effect of SPTBN1 silencing in the progression of primary osteoporosis both *in vitro* and *in vivo*. Taken together, these results suggested that SPTBN1 suppressed primary osteoporosis by facilitating the proliferation, differentiation, and inhibition of apoptosis in osteoblasts via the TGF-β/Smad3 and STAT1/Cxcl9 pathways. Besides, overexpression of SPTBN1 promoted the formation of blood vessels in bone by regulating the expression of VEGF. This study, therefore, provided SPTBN1 as a novel therapeutic target for osteoporosis.

## Introduction

Osteoporosis is a skeletal disorder characterized by compromised bone strength and predisposes a person to an increased risk of fracture, and is becoming an increasingly serious health challenge worldwide (Aspray and Hill, 2019). Osteoporosis often occurs in postmenopausal women and the senile population and is associated with increased mortality and morbidity globally (Miller, 2016). Generally, patients with osteoporosis are usually treated with calcium supplements and hormone replacement therapy (Kling et al., 2014; Khosla and Hofbauer, 2017). Although fragility fractures cause severe problems, available therapies for osteoporosis are unsatisfactory as they are associated with adverse side effects or poor patient compliance (Kerschan-Schindl, 2016). Therefore, understanding the pathogenesis of osteoporosis can extend knowledge and even contribute to the identification of efficient and sensitive therapeutic targets.

Spectrins, are a group of large, flexible, and highly conserved proteins containing α-β dimers that are connected head-to-head to form the canonical heterotetrameric spectrin structure (Marchesi and Steers, 1968; Derbala et al., 2018). βII-spectrin (SPTBN1), a member of the spectrin family, is an actin cross-linked molecular skeleton protein, which plays a crucial role in the arrangement of transmembrane proteins and organelle tissues (Susuki and Zollinger, 2018). Recently, several studies have reported that SPTBN1 is involved in the progression or prognosis of human diseases. For instance, Zhi et al. (2015) demonstrated that SPTBN1 inhibited the development of hepatocellular carcinoma via modulating the expression of Wnt inhibitor Kallistatin to intervene in the Wnt signaling pathway. Chen et al. (2020) reported that SPTBN1 can prevent the progression of epithelial ovarian cancer through the SOCS3-mediated blockade of the JAK/STAT3 signaling pathway. Besides, SPTBN1 has been implicated in the development of osteoporotic fracture and bone mineral density (BMD). For example, our recent system-level study based on genome-wide association studies of osteoporosis indicated that SPTBN1 associated with BMD in females (Chen et al., 2016). Wang et al. (2009) conducted a two-stage case-control association study with 1046 patients with the non-traumatic vertebra, hip, or distal radius fractures and 2303 healthy controls, and indicated that SPTBN1 was a susceptibility genetic loci for osteoporotic fracture in postmenopausal Chinese women (Wang et al., 2012). These reports suggest that SPTBN1 may contribute to the progression of osteoporosis. However, the specific functions and mechanisms of SPTBN1 in osteoporosis have not been reported.

Osteoblasts are the major bone-making cells that mature from mesenchymal progenitor cells and pre-osteoblasts, and produce the bone matrix during bone development (Ducy et al., 2000; Long, 2011). Pre-osteoblasts are often characterized by specific expression of transcription factors such as runt-related transcription factor 2 (Runx2) and Osterix (Osx) (Li N. et al., 2015), while osteoblasts are often characterized by specific expression of Osteocalcin (Ocn) (Zoch et al., 2016). The bone homeostasis *in vivo* depends on the balance between osteoclast absorption and osteoblast formation. Osteoporosis occurs when there is a decrease in osteoblast formation and an increase in osteoclast absorption (Chen et al., 2018). To identify mechanisms of SPTBN1 in osteoporosis, we explored pathways related to SPTBN1 by STRING website and found that SPTBN1 may associate with TGF-β/Smad3 and STAT1/Cxcl9 signaling pathways (Supplementary Figures 1A,B). Previous studies have indicated that TGF-β/Smad3 and STAT1/Cxcl9 signaling pathways are involved in the differentiation and functions of osteoblasts (Black and Rosen, 2016; Huang et al., 2016). Thus, SPTBN1 may regulate osteoblast by TGF-β/Smad3 and STAT1/Cxcl9 signaling pathways in osteoporosis.

Besides, bone is a highly vascularized form of connective tissue and blood vessels are essential for bone development, regeneration, and remodeling (Kanczler and Oreffo, 2008; Filipowska et al., 2017). The process of angiogenesis requires the participation of different growth and differentiation factors. The explore of pathways related to SPTBN1 by STRING website also indicated that SPTBN1 may associated with VEGF, which is very essential for angiogenesis in developing mature bone tissue (Liu and Olsen, 2014). Therefore, SPTBN1 may contribute to angiogenesis through regulating VEGF in osteoporosis.

Our results firstly demonstrated that SPTBN1 could efficiently prevent the progression of primary osteoporosis through facilitating the proliferation and differentiation of osteoblast, inhibiting apoptosis of osteoblast via regulating TGF-β/Smad3, STAT1/Cxcl9 pathways, and promoting the formation of blood vessels in bone by modulating VEGF expression.

## Materials and Methods

### Animal Model

A total of 120 C57BL-6 mice (60 male and 60 female) were purchased from the Guangdong Experimental Animal Center (Foshan, Guangdong, China), and kept at 20 ± 2°C, 12 h light/dark cycle and with approximately 50–60% humidity. For the senile osteoporosis model (*n* = 12), 9 months old mice were considered the senile osteoporosis group, and 3 months old mice were regarded as the young group. For the ovariectomized mice model (*n* = 12), mice were divided into the OVX group (ovariectomized) or sham group (sham surgery) which was performed as previously described (Li et al., 2019). To explore the function of SPTBN1, mice in the senile osteoporosis group and OVX group were intramedullary injected with the adeno-associated virus (AAV) used to silence SPTBN1 (si-SPTBN1) or SAM virus utilized to the overexpression of SPTBN1 (OE-SPTBN1) and corresponding negative controls (si-NC and OE-NC) into the right side of the femur. All animal experiments were performed according to the Guide for the Care and Use of Laboratory Animals published by the US National Institute of Health and this study was approved by Southern Medical University.

### Detection and Analysis of Micro-CT

One month after the first intramedullary injection, mice were anesthetized with pentobarbital and subsequently put to death by dislocation of cervical vertebra, and blood vessels were rinsed by 20 ml 100 μl/ml heparin from the left ventricle using Intravenous infusion needle. Subsequently, mice were fixed by 4% paraformaldehyde (PFA), perfused with contrast agent and placed at 4°C for 1–2 days. Next, bilateral femurs were harvested from the lower abdomen without fat and muscle and fixed by 4% PFA following decalcification for 3–4 weeks. Then, bilateral femurs were analyzed by micro-CT. Micro-CT imaging was performed using LathetaLCT-200 (Hitachi Aloka, Tokyo, Japan) *in vivo*. The 360-degree scanning of femurs *in vivo* was performed using the following parameters: 160 kV, 500 mA, a resolution of 10.44, and the Inveon Research Workplace 2.2 software was used for reconstruction of the 3D images. Micro-CT imaging *in vitro* was performed using Skyscan 1176 (Bruker MicrocT, Kontich, Belgium) in the femurs with the region of interest (ROI): 1 mm proximal to the epiphyseal plate and 0.5 mm in length. Finally, trabecular microarchitecture was evaluated by bone volume fraction (BV/TV), trabecular thickness (Tb. Th), trabecular number (Tb. N), and trabecular separation (Tb. Sp) according to published methods (Li et al., 2017).

### Immunohistochemistry (IHC) Assay

The IHC assay for the detection of SPTBN1, Ocn, Osx, and Runx2 was performed. In brief, the femur tissues were fixed with 10% formalin, embedded in paraffin, and sectioned. The slides were incubated with antibodies against SPTBN1 (Abcam, Cambridge, MA, United States), Ocn (Abcam), Osx (Abcam), Runx2 (Abcam), and VEGF (Abcam) at 4°C overnight, stained with Diaminobenzidine (DAB) and counterstained with hematoxylin. The representative images were captured using a light microscope.

### Tissue Alkaline Phosphatase (ALP)/Tartrate-Resistant Acid Phosphatase (TRAP) Staining Assay

The femur tissues were harvested and fixed with periodate-lysine-paraformaldehyde (PLP) fixative containing 0.075 M lysine, 2% PFA and 0.01 M sodium periodate solution (pH 7.4) at 4°C for 24 h. The femur tissues were decalcified according to the protocol described previously (Mori et al., 1988). Subsequently, the tissues were dehydrated by a graded series of alcohols and embedded in paraffin wax. The femurs were cut longitudinally at 5 μm thickness using a rotary microtome and processed for ALP and TRAP staining by the TRAP/ALP double staining kit (WAKO, Chuo-ku, Osaka, Japan) as previously described (Potu et al., 2009).

### H&E Staining Assay

H&E staining was performed as previously described (Liu et al., 2019). Briefly, the femur tissues were collected and fixed with 4% PFA at 4°C, dehydrated with gradient alcohol, cleared in xylene, embedded, and sliced to 4 μm in thickness. Then the slices were dyed in hematoxylin, washed away with running water, and dyed with 1% eosin. The slides were washed again with running water, dehydrated, cleared, blocked with neutral gum, and dried for 3 days. Finally, the femur tissues were photographed under a light microscope.

### Cell Culture

Mouse pre-osteoblast MC3T3-E1 cells (CRL-2594) were purchased from the American Type Culture Collection (ATCC) cell bank and cultured with 1640 medium supplemented with 10% fetal bovine serum (FBS) (Gibco, Rockville, MD, United States) at 37°C with 5% CO_2_. To induce cell differentiation, a differentiation medium containing 10% FBS with 50 μg/ml ascorbic acid and 4 mM β-glycerol phosphate was added when cells reached 80% confluence.

### Cell Transfection

Lentivirus (LV) used to silence SPTBN1 (Si-SPTBN1), SAM virus utilized to the overexpression of SPTBN1 (OE-SPTBN1), and their corresponding controls (Si-NC and OE-NC) were transfected into MC3T3-E1 cells using Lipofectamine 2000 kit (Invitrogen, Thermo Fisher Scientific, Shanghai, China) according to the manufacturer’s instructions. After transfection for 48 h, the transfection efficiency was evaluated by qRT-PCR and the optimal silencing or overexpressing lentivirus collected for subsequent experiments. The sequences for si-SPTBN1 were as follows: 200: 5′-CCGGCCTCGTATTGATGACATCTTTCTCGAGAAAGATGT CATCAATACGAGGTTTTT-3′; 201: 5′-CCGGGCCAGAAAT CTGCACAGTAAACTCGAGTTTACTGTGCAGATTTCTGGC TTTTT-3′; 202: 5′-CCGGCGCTTCCAGATCCAGGATATTCTC GAGAATATCCTGGATCTGGAAGCGTTTTT-3′.

### CCK-8 Assay

To measure cell viability, approximately 5 × 10^3^ cells MC3T3-E1 cells were seeded into 96-well plates. Hundred μL of CCK-8 solution (Dojindo Molecular Technologies, Japan) was added to each well and incubated for 24, 48, 72, and 96 h, respectively. Finally, the optical density (OD) at 450 nm was detected using a microplate reader (Bio Tek Instruments, Winooski, VT, United States).

### Apoptosis Analysis

Cell apoptosis was evaluated by flow cytometry after treatment with the Annexin V: FITC Apoptosis Detection Kit II (BD Biosciences, San Jose, CA, United States) according to the manufacturer’s instructions. Briefly, cells were centrifuged and resuspended with binding buffer. Then, cells were incubated with 5 μL of Annexin V-FITC for 15 min followed by 10 μL of propidium iodide (PI) for 5 min. Subsequently, the apoptosis rate was evaluated by Flow cytometry (BD Biosciences).

### ALP Activity Assay

After transfection with different lentivirus, ALP activity of MC3T3-E1 cells was measured by an ALP activity kit according to the manufacturer’s protocol (Beyotime, Shanghai, China). The absorbance was determined at 405 nm.

### Alizarin Red Staining (ARS) Assay

Alizarin red staining assay was performed to detect osteoblast calcification. Briefly, MC3T3-E1 cells were seeded into 12 well plates at a density of 5 × 10^4^ cells/well and cultured for 24 h for transfection. After transfection for 48, the cells were fixed with 95% ethanol for 10 min, washed thrice with PBS, covered and stained using Alizarin Red S staining solution (Cyagen, Guangzhou, Guangdong, China) for 15 min. Finally, the cells were rinsed with PBS and photographed under a microscope.

### ELISA Assay

The culture supernatant of MC3T3-E1 cells were centrifuged at 3000 rpm/min at 4°C for 25 min. The supernatant was collected and the expression levels of Cxcl9 detected using ELISA kits (ab203364, Abcam, Shanghai, China) according to the manufacturer’s instructions.

### qRT-PCR

Total RNA of the cultured cells was extracted using TRIzol reagent (Invitrogen, Carlsbad, CA, United States). Approximately 1.2 μg RNA was reverse-transcribed into cDNA with the TaqMan MicroRNA Reverse Transcription Kit, and quantitative RT-PCR was performed using the ABI Prism 5700 Sequence Detection System (Forest City, CA, United States). The relative expression change of SPTBN1 was analyzed by the 2^–Δ^ ^Δ^ ^Ct^ method with GAPDH as the internal reference. The primers used were as follows: SPTBN1 forward primer: 5′-GAGTTGCAGAGGACATCCAGC-3′, reverse primer: 5′-ATTGACCCACTTGGTGAAGGTC-3′; GAPDH forward primer: 5′-ACCACA GTCCATGCCATCAC-3′, reverse primer: 5′-TCCACCACCCTGTTGCTGTA-3′.

### Western Blot

Total protein was isolated using RIPA lysis (Trans-Gen Biotech) according to the manufacturer’s instructions. Approximately, an equal amount of protein was separated using 12% SDS-PAGE and transferred onto PVDF membranes. After blocking with 5% non-fat milk for 1h at room temperature, the membranes were incubated with primary antibodies against CyclinE1 (ab33911, Abcam), Cleaved Caspase 3 (ab13847, Abcam), Runx2 (ab192256, Abcam), Osx (ab209484, Abcam), Ocn (ab133612, Abcam), SPTBN1 (ab72239, Abcam), TGF-β (ab215715, Abcam), p-STAT1 (ab109461, Abcam), p-SMAD3 (ab52903, Abcam), and β-actin (KM9001, Sungene Biotech, Tianjin, China). β-actin was served as the internal reference of Western Blot in this study. Subsequently, the membranes were incubated with HRP-labeled secondary antibody (1:5000) for 1 h. Finally, the detection of protein bands was performed using an enhanced chemiluminescence (ECL) kit (Santa Cruz Biotechnology, Dallas, TX, United States) according to the manufacturer’s instructions, and the bands of interest were visualized using a Bio-Rad imaging system (Bio-Rad Laboratories, Mississauga, ON, United States).

### RNA Sequencing and Analysis of Differential Expressed Genes

Total RNA was extracted from Si- SPTBN1 and Si-NC differentiated MC3T3-E1 cells by TRIzol (Invitrogen). After enriching mRNA, samples were sequenced by Gene *Denovo* Biotechnology Co. (Guangzhou, Guangdong, China) using Illumina HiSeq2500. Next, the gene abundance was quantified by StringTie software (v1.3.1) (Pertea et al., 2015). Subsequently, analysis of differential expressed genes between two different groups was performed using DESeq2 software (Love et al., 2014). Genes with false discovery rate (FDR) < 0.05 and an absolute fold change more than 2 were identified as differential expressed genes.

### Gene Ontology (GO) and KEGG Pathway Enrichment Analysis

Gene Ontology enrichment for differentially expressed genes (DEGs) was analyzed by GOseq R package (Young et al., 2010). For KEGG pathway enrichment of DEGs, the KOBAS software program was utilized as described previously (Mao et al., 2005).

### Statistical Analysis

All data were presented as mean ± standard deviation (SD). Data analysis was performed by SPSS version 17.0 software. All experiments were repeated three times. The difference between the two groups was determined by Student’s *t*-test. Multiple group comparisons were analyzed using one-way analysis of variance (ANOVA). *P* < 0.05 was considered statistically significant.

## Results

### The Expression of SPTBN1 Is Significantly Downregulated in Primary Osteoporosis Mice

**FIGURE 1:**
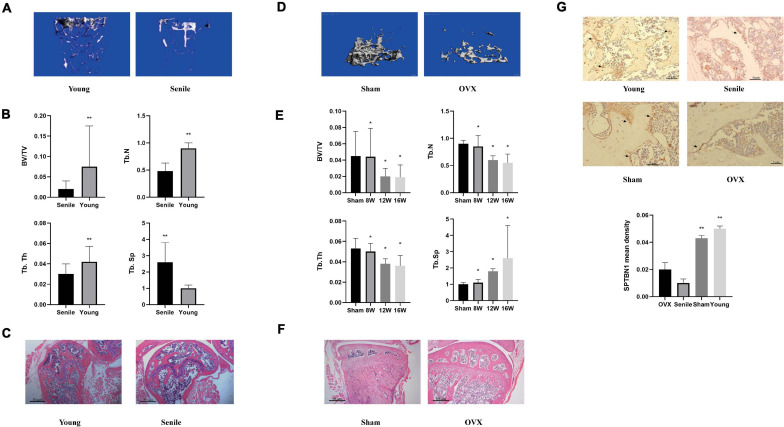
SPTBN1 was significantly downregulated in a primary osteoporosis mouse model. **(A–C)** The establishment of the senile osteoporosis mice model (Young group and Senile group). **(A)** Micro-CT imaging and 3D reconstruction of metaphyseal trabecular bone of the distal femur. **(B)** Morphometry of trabecular BV/TV, Tb. Th, Tb. N, and Tb. Sp based on Micro-CT imaging (*n* = 8). **(C)** Representative photomicrographs of hematoxylin-eosin (HE) staining in sections of the distal femur (*n* = 8). **(D–F)** The establishment of the ovariectomized osteoporosis mice model (Sham group and OVX group). **(D)** Micro-CT imaging and 3D reconstruction of metaphyseal trabecular bone of the distal femur. **(E)** Morphometry of trabecular BV/TV, Tb. Th, Tb. N, and Tb. Sp based on Micro-CT imaging (*n* = 8). **(F)** Representative photomicrographs of HE staining of SPTBN1 in sections of the distal femur (*n* = 8). The magnification of photomicrographs of HE staining was 20×. **(G)** Immunohistochemistry staining for SPTBN1 in sections of the distal femur of the OVX group, Sham group, Senile group, and Young group. The mean density of the SPTBN1 positive cells was calculated as integrated optical density (IOD) per area of positive cells (*n* = 8). The magnification of photomicrographs of immunohistochemistry was 40×, and arrows indicated SPTBN1 positive cells stained in brown. Scale bar, 5 μm for all. ^∗^*P* < 0.05, ^∗∗^*P* < 0.01 vs. control group.

To explore the specific roles and underlying mechanisms of SPTBN1 in primary osteoporosis, the primary osteoporosis mice models including spontaneous senile osteoporosis mice model and ovariectomized (OVX) mice model were established. For the spontaneous senile osteoporosis mice model, the representative images of Micro-CT imaging of the metaphyseal trabecular bone in the distal femur between the young group and senile group are shown in Figure 1A. The images indicated that the number of bone trabecula was significantly reduced, and the gap was widened in the senile group compared with the young group. The BV/TV (*p* < 0.01), Tb. N (*p* < 0.01), Tb. Th (*p* < 0.01) in the senile group was significantly decreased compared with the young group, while Tb. Sp (*p* < 0.01) was significantly increased in the senile group (Figure 1B). The representative photomicrographs of H&E staining in sections of the distal femur between the senile group and young group are shown in Figure 1C. The results indicated that there was a significant increase in adipose tissue and fracture tendency in the senile group compared with the young group (Figure 1C). For the osteoporosis mice model, representative images of Micro-CT imaging are shown in Figure 1D. The results suggested that the number of bone trabecula was significantly reduced, and the gap was widened in the OVX group compared with the sham group. Similarly, after OVX at different times such as 8, 12, or 16 weeks, the BV/TV (*p* < 0.05), Tb. N (*p* < 0.05), Tb. Th (*p* < 0.05) were significantly decreased in the OVX group compared with the sham group, and Tb. Sp was increased (*p* < 0.05) (Figure 1E). The representative photomicrographs of H&E staining are shown in Figure 1F, indicating that there was a significant increase in adipose tissue and fracture tendency in the OVX group compared with the sham group. These results suggested that the two primary osteoporosis mice models were successfully established and could be used for subsequent experiments. Further, an IHC assay was performed to evaluate the relative expression of SPTBN1 in the distal femur from different groups. The results showed that the mean density of SPTBN1 positive cells was significantly decreased both in the OVX group and senile group compared with the respective control groups (Figure 1G). These results suggested a potential protective role of SPTBN1 in the progression of primary osteoporosis.

### SPTBN1 Silence Exacerbates the Progression of Primary Osteoporosis

To further determine the function of SPTBN1 in primary osteoporosis, the SPTBN1 silencing and overexpression assays were performed. Here, primary osteoblast MC3T3-E1 cells were transfected with AAV or SAM carrying three different silencing sequences or overexpression sequence to optimize the most effective sequences. The representative transfection fluorescence after 48 h of transfection with different si-SPTBN1 sequences including 200, 201, and 202 are shown in Supplementary Figure 2A. Meanwhile, qRT-PCR and western blot results indicated that, when transfected with si-SPTBN1 202, both the mRNA and protein expression of SPTBN1 were the lowest compared with transfection with si-SPTBN1 200 and 210 (Supplementary Figures 2B,C). Similarly, the representative transfection fluorescence after 48 h of transfection with different OE-SPTBN1 sequences including 6164, 6165, and 6166 are shown in Supplementary Figure S2D. The expression of SPTBN1 in the OE-SPTBN1 6166 group was the highest compared with the other two groups (Supplementary Figures 2E,F). These results suggested that si-SPTBN1 (202) and OE-SPTBN1 (6166) achieved the best efficiency and were selected for subsequent experiments.

**FIGURE 2:**
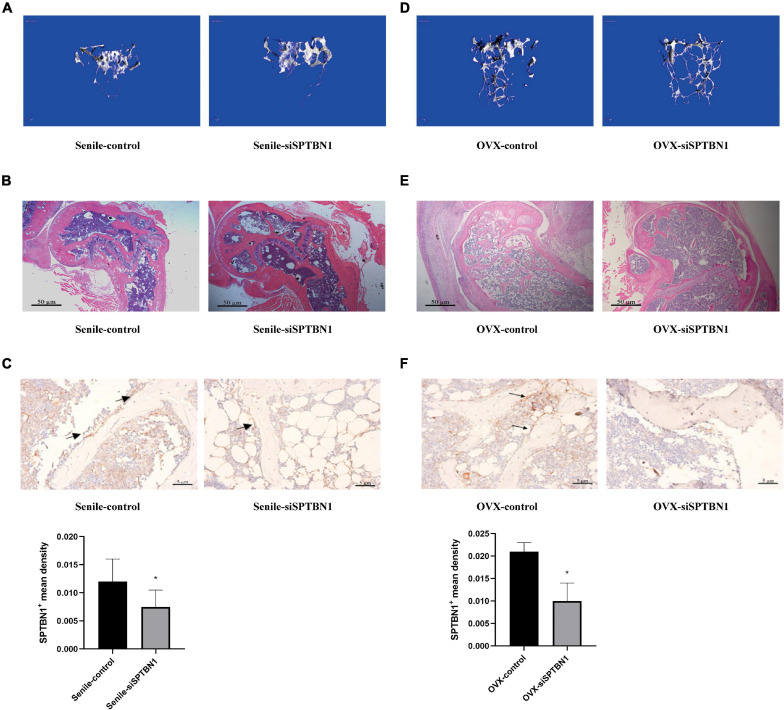
The effect of si-SPTBN1 in primary osteoporosis mice. **(A–C)** The effect of si-SPTBN1 in senile osteoporosis mice. **(A)** Micro-CT imaging and 3D reconstruction of metaphyseal trabecular bone of the distal femur. **(B)** Representative photomicrographs of HE staining in sections of the distal femur (*n* = 8). **(C)** Immunohistochemistry staining for SPTBN1 in sections of the distal femur. The mean density of the SPTBN1 positive cells was calculated as integrated optical density (IOD) per area of positive cells (*n* = 8). **(D–F)** The effect of si-SPTBN1 in ovariectomized osteoporosis mice. **(D)** Micro-CT imaging and 3D reconstruction of metaphyseal trabecular bone of the distal femur. **(E)** Representative photomicrographs of HE staining in sections of the distal femur (*n* = 8). **(F)** Immunohistochemistry staining for SPTBN1 in sections of the distal femur. The mean density of the SPTBN1 positive cells was calculated as integrated optical density (IOD) per area of positive cells (*n* = 8). The magnification of photomicrographs of HE staining was 20×, while the magnification of photomicrographs of immunohistochemistry was 40×. Arrows indicate the SPTBN1 positive cells stained in brown. Scale bar, 5 μm for all. ^∗^*P* < 0.05 vs. control group.

To confirm the protective role of SPTBN1 in primary osteoporosis, the marrow cavity of the left femur of senile and OVX mice was injected with control lentivirus, and the marrow cavity of the right femur was injected with si-SPTBN1 lentivirus. The representative images of Micro-CT imaging and H&E staining in the femur of si-SPTBN1 injected senile mice are shown in Figures 2A,B. These images indicated that in the si-SPTBN1 group, the number of trabecula decreased, the trabecula were thinner, the gap was widened, and osteoporosis was aggravated compared with the control group (Table 1). Meanwhile, IHC assay indicated that si-SPTBN1 significantly decreased the SPTBN1 mean density in the femur of senile mice compared with the control group (Figure 2C). The representative images of Micro-CT and H&E staining in the femur of si-SPTBN1 injected OVX mice are shown in Figures 2D,E. The images showed that in the si-SPTBN1 group, the number of trabecula was decreased, the trabecula were thinner, the gap wider and osteoporosis was aggravated compared with the control group (Table 2). Besides, the SPTBN1 mean density in the OVX si-SPTBN1 group was significantly decreased compared with the OVX control group (Figure 2F). These results indicated that the downregulation of SPTBN1 could significantly exacerbate primary osteoporosis.

**TABLE 1:** Micro-CT scanning parameter comparison of cancellous and cortical bone between senile control and senile si-SPTBN1 mice.

	Senile control	Senile si-SPTBN1	*P*-value
CORTICAL.BONE. BMD(mg HA/ccm)	540.57 ± 62.18	460.07 ± 59.34	0.008
CANCELLOUS.BONE. BMD(mg HA/ccm)	92.06 ± 16.12	84.70 ± 19.46	0.369
TOTAL.BONE.BMD (mg HA/ccm)	419.89 ± 61.20	361.95 ± 46.33	0.028
BV.TV/mm	14.31 ± 3.19	10.95 ± 2.60	0.073
TB.N/mm	1.49 ± 0.38	1.00 ± 0.21	0.022
TB.TH/mm	0.13 ± 0.02	0.10 ± 0.01	0.025
TB.SP/mm	0.67 ± 0.16	0.80 ± 0.15	0.178

*P < 0.05 was considered statistically significant.

**TABLE 2:** Micro-CT scanning parameter comparison of cancellous and cortical bone between OVX control and OVX si-SPTBN1 mice.

	OVX control	OVX si-SPTBN1	*P*-value
CORTICAL.BONE. BMD(mg HA/ccm)	650.57 ± 39.89	570.17 ± 47.66	<0.001
CANCELLOUS.BONE. BMD(mg HA/ccm)	107.26 ± 26.80	92.51 ± 18.82	0.171
TOTAL.BONE.F.BMD (mg HA/ccm)	519.89 ± 61.20	456.28 ± 44.59	0.016
BV.TV/mm	13.82 ± 2.64	11.14 ± 1.58	0.058
TB.TH/mm	0.14 ± 0.03	0.11 ± 0.02	0.039
TB.N/mm	1.53 ± 0.27	1.05 ± 0.22	0.007
TB.SP/mm	0.64 ± 0.15	0.74 ± 0.14	0.253

*P < 0.05 was considered statistically significant.

### Upregulation of SPTBN1 Inhibits the Progression of Primary Osteoporosis

**FIGURE 3:**
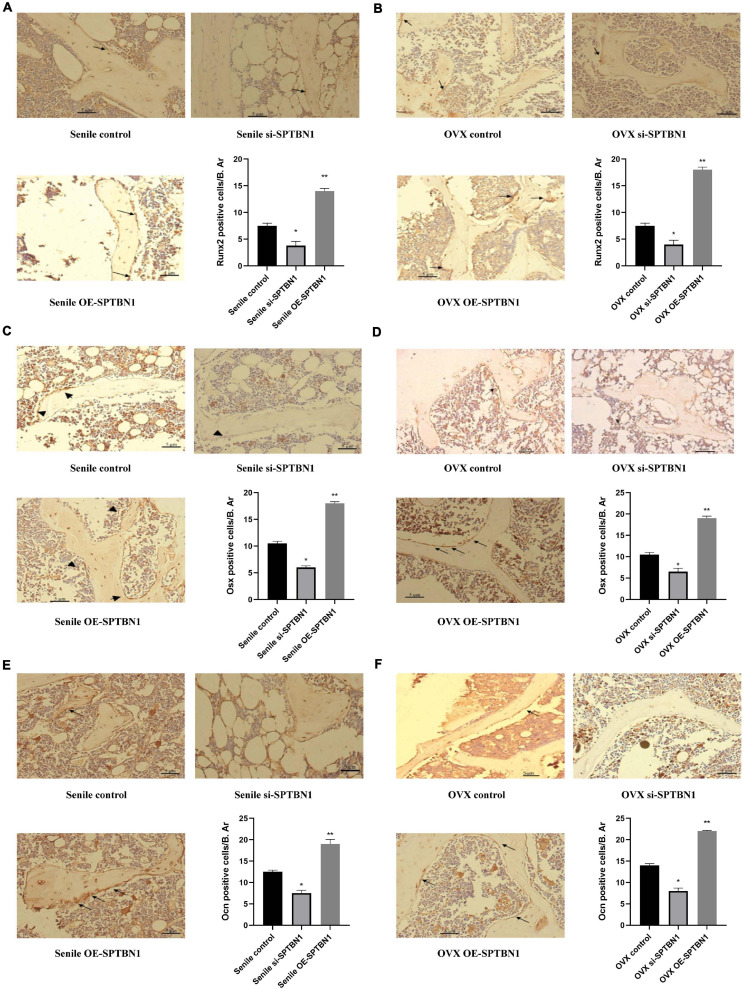
The effect of OE-SPTBN1 in primary osteoporosis mice. **(A–C)** The effect of OE-SPTBN1 in senile osteoporosis mice. **(A)** Micro-CT imaging and 3D reconstruction of metaphyseal trabecular bone of the distal femur. **(B)** Representative photomicrographs of HE staining in sections of the distal femur (*n* = 8). **(C)** Immunohistochemistry staining for SPTBN1 in sections of the distal femur (*n* = 8). **(D–F)** The effect of OE-SPTBN1 in ovariectomized osteoporosis mice. **(D)** Micro-CT imaging and 3D reconstruction of metaphyseal trabecular bone of the distal femur. **(E)** Representative photomicrographs of HE staining in sections of the distal femur (*n* = 8). **(F)** Immunohistochemistry staining for SPTBN1 in sections of the distal femur. The mean density of the SPTBN1 positive cells was calculated as integrated optical density (IOD) per area of positive cells (*n* = 8). The magnification of photomicrographs of HE staining was 20×, while the magnification of photomicrographs of immunohistochemistry was 40×. Arrows indicate the SPTBN1 positive cells stained in brown. Scale bar, 5 μm for all. ^∗^*P* < 0.05 vs. control group.

The marrow cavity of the left femur of senile and OVX mice was injected with the control lentivirus, and the marrow cavity of the right femur was injected with OE-SPTBN1 lentivirus. The representative images of Micro-CT and H&E staining in the femur of OE-SPTBN1 injected senile mice are shown in Figures 3A,B. The images showed that the number of trabecula was increased, the trabecula were thicker, the gap narrowed, and osteoporosis was attenuated in the OE-SPTBN1 group compared with the control group (Table 3). Figure 3C indicated that OE-SPTBN1 significantly increased the SPTBN1 mean density in the femur of senile mice compared with the control group. Besides, the representative images of Micro-CT and H&E staining in the femur of OE-SPTBN1 injected OVX mice are shown in Figures 3D,E. The images showed that the number of trabecula was increased, the trabecula were thicker, the gap narrowed, and osteoporosis was attenuated in the OE-SPTBN1 group compared with the control group (Table 4). The SPTBN1 mean density in the OVX OE-SPTBN1 group was significantly increased compared with the OVX control group (Figure 3F). These results suggested that the upregulation of SPTBN1 could significantly inhibit primary osteoporosis.

**TABLE 3:** Micro-CT scanning parameter comparison of cancellous and cortical bone between senile control and senile OE-SPTBN1 mice.

	Senile control	Senile si-SPTBN1	*P*-value
CORTICAL.BONE. BMD(mg HA/ccm)	485.44 ± 51.48	531.84 ± 46.95	0.039
CANCELLOUS.BONE. BMD(mg HA/ccm)	84.79 ± 9.95	94.00 ± 11.14	0.054
TOTAL.BONE.F.BMD (mg HA/ccm)	380.15 ± 51.96	436.16 ± 42.48	0.012
BV.TV/mm	22.19 ± 15.77	26.68 ± 16.04	0.497
TB.TH/mm	0.12 ± 0.01	0.10 ± 0.03	0.028
TB.N/mm	2.02 ± 1.64	3.62 ± 3.67	0.182
TB.SP/mm	0.42 ± 0.20	0.39 ± 0.21	0.752

*P < 0.05 was considered statistically significant.

**TABLE 4:** Micro-CT scanning parameter comparison of cancellous and cortical bone between OVX control and OVX OE-SPTBN1 mice.

	OVX control	OVX OE-SPTBN1	*P*-value
CORTICAL.BONE. BMD(mg HA/ccm)	569.98 ± 25.22	626.38 ± 48.58	0.003
CANCELLOUS.BONE. BMD(mg HA/ccm)	100.25 ± 19.78	116.73 ± 36.60	0.204
TOTAL.BONE.F.BMD (mg HA/ccm)	443.79 ± 23.37	486.16 ± 53.71	0.026
BV.TV/mm	23.49 ± 16.75	28.41 ± 16.81	0.315
TB.TH/mm	0.13 ± 0.03	0.16 ± 0.08	0.186
TB.N/mm	2.20 ± 1.76	3.82 ± 3.54	0.05
TB.SP/mm	0.36 ± 0.20	0.34 ± 0.20	0.699

*P < 0.05 was considered statistically significant.

### SPTBN1 Prevents Primary Osteoporosis by Promoting Osteoblasts Proliferation and Differentiation

The cellular mechanisms of SPTBN1 induced inhibitory effects in primary osteoporosis were explored and ALP staining performed in the metaphysis of the distal femur of mice with different treatments. The active site of ALP was stained blue and represented osteoblasts. The representative images of ALP staining in the senile group and OVX group transfected with negative, si-SPTBN1, or OE-SPTBN1 are shown in Supplementary Figures 3A,B. The quantitation data revealed that si-SPTBN1 significantly decreased the ALP positive cells (osteoblasts) per bone area both in the senile mice and OVX mice compared with the control group, while OE-SPTBN1 significantly increased the ALP positive cells (osteoblasts) per bone area both in the senile mice and OVX mice compared with the control (Supplementary Figures 3C,D). These results indicated that SPTBN1 inhibited primary osteoporosis by promoting osteoblast proliferation.

**FIGURE 4:**
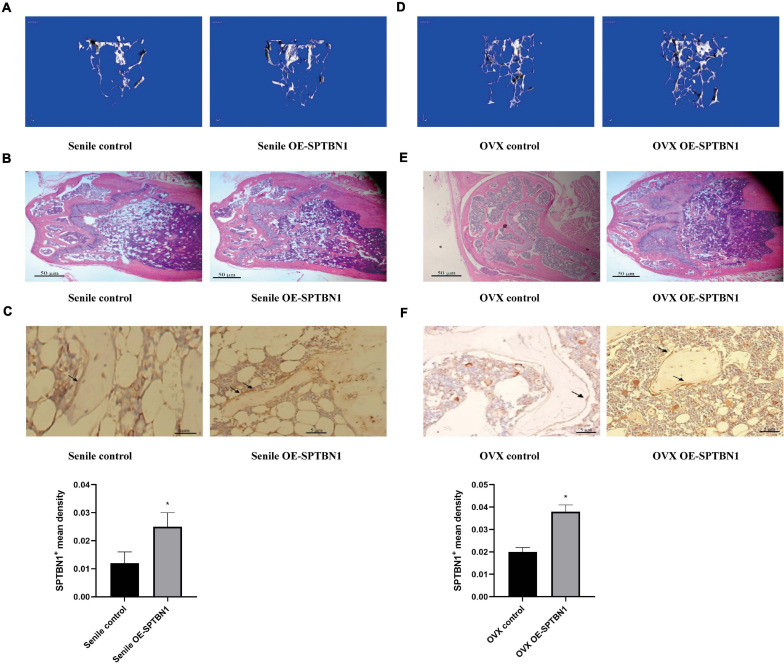
The effect of si-SPTBN1 and OE-SPTBN1 in osteoblast differentiation in the distal femur of primary osteoporosis mice. **(A,B)** Immunohistochemistry staining for Runx2 in the distal femur of si-SPTBN1 or OE-SPTBN1 transfected senile **(A)** and OVX **(B)** osteoporosis mice. **(C,D)** Immunohistochemistry staining for Osx in the distal femur of si-SPTBN1 or OE-SPTBN1 transfected senile **(C)** and OVX **(D)** osteoporosis mice. **(E,F)** Immunohistochemistry staining for Ocn in the distal femur of si-SPTBN1 or OE-SPTBN1 transfected senile **(E)** and OVX **(F)** osteoporosis mice. The mean density of the respective positive cells was calculated as integrated optical density (IOD) per area of positive cells (*n* = 8). The magnification of photomicrographs of immunohistochemistry was 40×. Arrows indicate the Runx2, Osx, or Ocn positive cells stained in brown. Scale bar, 5 μm for all. *P < 0.05 vs. control group.

Immunohistochemistry assay was performed to evaluate the expression of osteoblast differentiation-related makers such as Runx2, Osx, and Ocn in the metaphysis of the distal femur of the mice with different treatments. The representative images of IHC are shown in Figures 4A–F. The results indicated that downregulation of SPTBN1 significantly decreased Runx2 (Figures 4A,B), Osx (Figures 4C,D), and Ocn (Figures 4E,F) positive cells both in the senile mice and OVX mice compared with the control group, while upregulation of SPTBN1 significantly increased Runx2 (Figures 4A,B), Osx (Figures 4C,D), and Ocn (Figures 4E,F) positive cells in both the senile mice and OVX mice compared with the control group. These results suggested that SPTBN1 inhibited primary osteoporosis by promoting osteoblast differentiation.

Besides, the TRAP staining assay was used to evaluate the effect of si-SPTBN1 and OE-SPTBN1 in osteoclast proliferation, and the active site of TRAP was stained purple and represented osteoclasts. The representative images of TRAP staining in the senile group and OVX group transfected with negative, si-SPTBN1, or OE-SPTBN1 are shown in Supplementary Figures 4A–D. The quantitation data indicated that si-SPTBN1 significantly increased the TRAP-positive cells both in the senile mice and OVX mice compared with the control group (Supplementary Figures 4A,C), while OE-SPTBN1 decreased the TRAP-positive cells in both the senile mice and OVX mice compared with the control group (Supplementary Figures 4B,D). All these results suggested that SPTBN1 inhibited primary osteoporosis by promoting proliferation and differentiation of osteoblasts, as well as inhibiting osteoclast proliferation.

### SPTBN1 Inhibits the Proliferation of MC3T3-E1 Cells by Inducing Apoptosis

**FIGURE 5:**
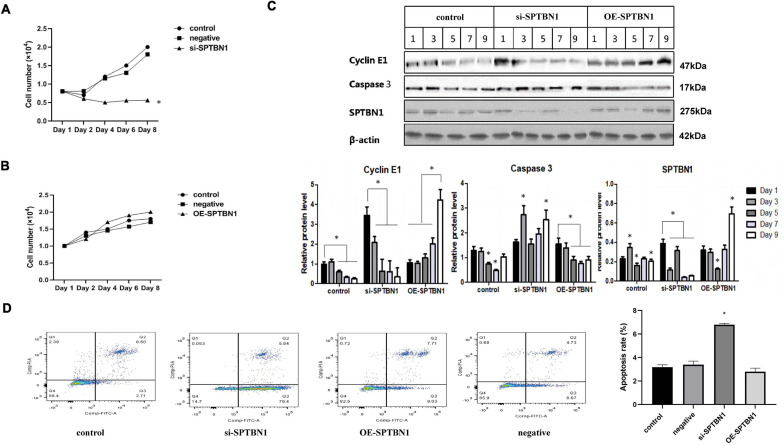
The effect of si-SPTBN1 and OE-SPTBN1 in the proliferation and apoptosis of MC3T3-E1 cells *in vitro*. **(A,B)** MC3T3-E1 cells transfected with si-SPTBN1 **(A)** or OE-SPTBN1 **(B)** and cultured at different times (1, 3, 5, and 7 days). The cell viability was evaluated by CCK-8 assay. **(C)** MC3T3-E1 cells transfected with OE-SPTBN1 or si-SPTBN1 and cultured at different times (1, 3, 4, 5, 7 and 9 days). The protein expression of SPTBN1, Cyclin, and Cleaved Caspase 3 was evaluated by Western blot, with β-actin as the internal reference. **(D)** MC3T3-E1 cells transfected with OE-SPTBN1 or si-SPTBN1 and cultured for 4 days. The apoptosis rate was evaluated by flow cytometry. *N* = 3, ^∗^*P* < 0.05 vs. control group.

To explore the specific role and mechanism of SPTBN1 in primary osteoporosis, primary osteoblast MC3T3-E1 cells were transfected with si-SPTBN1, OE-SPTBN1, and corresponding controls. CCK-8 assay indicated that si-SPTBN1 significantly decreased the cell viability of MC3T3-E1 cells compared with the control group (Figure 5A). Besides, OE-SPTBN1 significantly increased the cell viability of MC3T3-E1 cells (Figure 5B). si-SPTBN1 or OE-SPTBN1 transfected MC3T3-E1 cells were cultured for 1, 3, 5, 7, and 9 days and the protein expression of SPTBN1, Cyclin, and Cleaved Caspase 3 was evaluated by Western blot. The results indicated that OE-SPTBN1 significantly increased the protein expression of SPTBN1 and Cyclin but exhibited no significant change in Cleaved Caspase 3 expression. Besides, si-SPTBN1 significantly decreased the protein expression of SPTBN1, Cyclin, and Cleaved Caspase 3 (Figure 5C). The apoptosis rate of MC3T3-E1 cells at different treatments was evaluated by flow cytometry and the results showed that si-SPTBN1 significantly promoted cell apoptosis, while OE-SPTBN1 exhibited no significant change in cell apoptosis (Figure 5D). These results suggested that SPTBN1 inhibited the proliferation and induced apoptosis of MC3T3-E1 cells *in vitro*.

### SPTBN1 Promotes the Differentiation of MC3T3-E1 Cells

**FIGURE 6:**
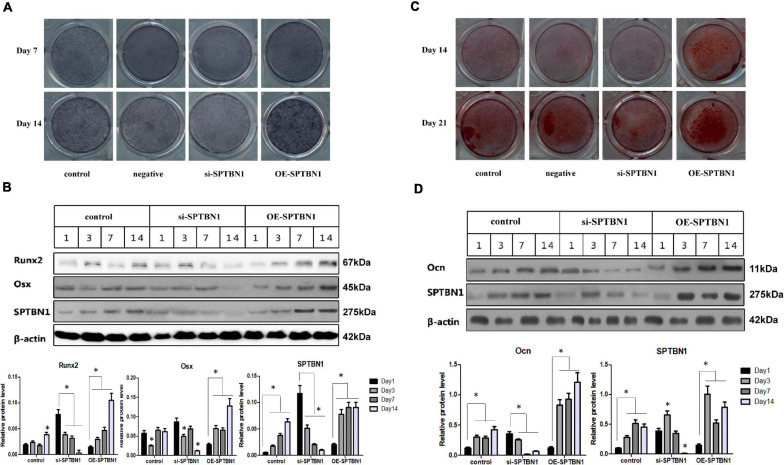
The effect of si-SPTBN1 and OE-SPTBN1 in osteoblast differentiation *in vitro*. **(A)** ATP staining on the 7th and 14th day of osteogenic induction culture before transfection of si-SPTBN1 or OE-SPTBN1 into MC3T3-E1 cells. **(B)** MC3T3-E1 cells transfected with si-SPTBN or OE-SPTBN1 for 1, 3, 5, 7, and 14 days. Protein expression of SPTBN1, Osx, and Runx2 was evaluated by Western blot analysis. **(C)** Alizarin red staining on the 14th and 21st days of osteogenic induction culture before transfection of si-SPTBN1 or OE-SPTBN1 into MC3T3-E1 cells. **(D)** MC3T3-E1 cells transfected with OE-SPTBN1 or si-SPTBN1 for 1, 3, 7, and 14 days. Protein expression of Ocn and SPTBN1 was evaluated by Western blot analysis, with β-actin as the internal reference. *N* = 3. ^∗^*P* < 0.05 vs. control group.

To investigate whether SPTBN1 affected the early or later differentiation of osteoblasts *in vitro*, MC3T3-E1 cells transfected with si-SPTBN1, OE-SPTBN1 or corresponding controls were induced using osteogenic induction medium at different times, including 7, 14, or 21 days. The representative images of ALP staining for MC3T3-E1 cells at days 7 and 14 are shown in Figure 6A. The results indicated that si-SPTBN1 significantly decreased the area of ALP positive expression at day 7 and 14 compared with the control group, while OE-SPTBN1 significantly increased the area of ALP positive expression at day 7 and 14. Meanwhile, si-SPTBN1 was found to significantly decrease the protein expression level of Runx2 and Osx during the early differentiation of MC3T3-E1 cells, while OE-SPTBN1 increased the protein expression of Runx2 and Osx (Figure 6B). These results suggested that the activity of SPTBN1 was positively correlated with the early differentiation of pre-osteoblasts. Besides, the representative images of Alizarin red staining for MC3T3-E1 cells at day 14 and 21 are shown in Figure 6C, indicating that si-SPTBN1 significantly decreased the area of Alizarin red expression at day 14 and 21 compared with the control group, while SO-SPTBN1 increased the area of Alizarin red expression at day 14 and 21 compared with the control group. Meanwhile, the results of Western blot analysis showed that OE-SPTBN1 significantly increased the protein expression of Ocn, while si-SPTBN1 decreased the protein expression of Ocn (Figure 6D). These results suggested that SPTBN1 could promote the differentiation and mineralization of pre-osteoblasts MC3T3-E1 cells *in vitro*.

### SPTBN1 Decreases the Activity of Smad3/TGF-β and STAT1/Cxcl-9 Signaling Pathways in MC3T3-E1 Cells

To further explore downstream target genes and pathways of SPTBN1, RNA sequencing was utilized. Results showed that 350 genes were upregulated while 151 genes were downregulated in SPTBN1-silenced MC3T3-E1 cells compared those in control cells (Supplementary Table 1). Further analysis found that silence of SPTBN1 induced the expression of Cxcl-9 (Supplementary Table 1). STAT1/Cxcl9 signaling pathway plays vital role in the differentiation and functions of osteoblasts as well as angiogenesis of the bone marrow (Huang et al., 2016). Moreover, the GO terms indicated that differently expressed genes in SPTBN1-silenced MC3T3-E1 cells enriched in biology processes including negative regulation of bone mineralization involved in bone maturation, bone morphogenesis, bone remodeling and so on (Supplementary Table 2). Further analysis of KEGG pathway enrichment for differently expressed genes in SPTBN1-silenced MC3T3-E1 cells demonstrated that enriched pathways were related to Rheumatoid arthritis, TNF signaling pathway, TGF-beta signaling pathway et al. (Supplementary Table 3). Previous studies have revealed that Smad3/TGF-β signaling pathway are associated with proliferation, invasion, differentiation, and apoptosis of osteoblasts (Huang et al., 2015). Therefore, SPTBN1 may suppress primary osteoporosis of osteoblasts through Smad3/TGF-β and STAT1/Cxcl-9 signaling pathways.

**FIGURE 7:**
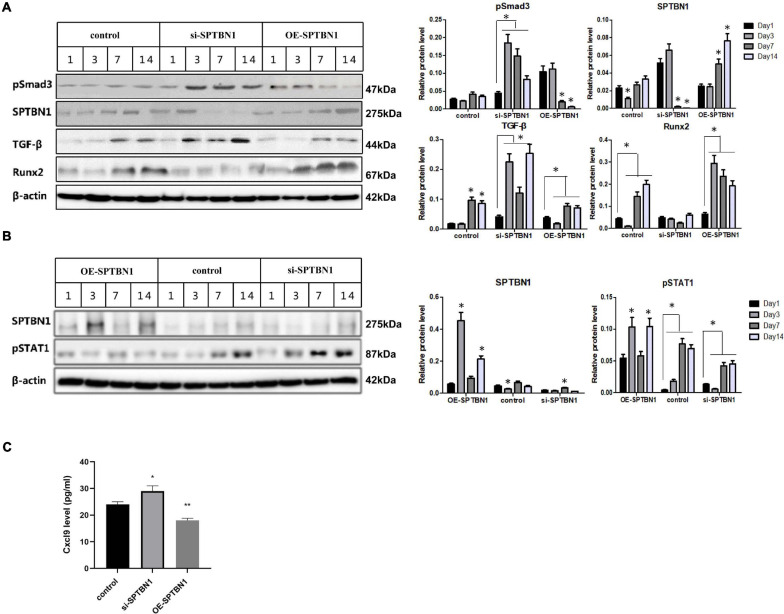
The effect of si-SPTBN1 and OE-SPTBN1 in the Smad3/TGF-β and STAT1/Cxcl-9 pathways *in vitro*. **(A)** MC3T3-E1 cells were transfected with si-SPTBN1 or OE-SPTBN1 for 1, 3, 5, 7, and 14 days, then the protein expression of SPTBN1, Runx2, TGF-β and p-Smad3 was evaluated by Western blot. **(B)** MC3T3-E1 cells were transfected with si-SPTBN1 or OE-SPTBN1 for 1, 3, 5, 7, and 14 days, then the protein expression of SPTBN1 and p-STAT1 was evaluated by Western blot. β-actin was considered as the internal reference. **(C)** The expression of Cxcl-9 was evaluated by ELISA assay. *N* = 3, ^∗^*P* < 0.05, ^∗∗^*P* < 0.01 vs. control group.

Next, we identified the effect of SPTBN1 in the activation of Smad3/TGF-β and STAT1/Cxcl-9 signaling pathways. Results of Western blot analysis indicated that si-SPTBN1 significantly increased the protein level of p-Smad3 and TGF-β, and decreased Runx2 expression; OE-SPTBN1 significantly decreased the protein expression level of p-Smad3 and TGF-β but increased Runx2 expression (Figure 7A). Moreover, si-SPTBN1 significantly increased the protein level of p-STAT1 (Figure 7B) and the release of Cxcl9 (Figure 7C), while OE-SPTBN1 decreased the protein expression level of p-STAT1 (Figure 7B) and the secretion of Cxcl9 (Figure 7C). These results suggested that SPTBN1 inhibited the activity of Smad3/TGF-β and STAT1/Cxcl-9 signaling pathways *in vitro*.

### SPTBN1 Suppresses Primary Osteoporosis by Inhibiting the Formation of Bone Microvessels via Reducing the Expression of VEGF

**FIGURE 8:**
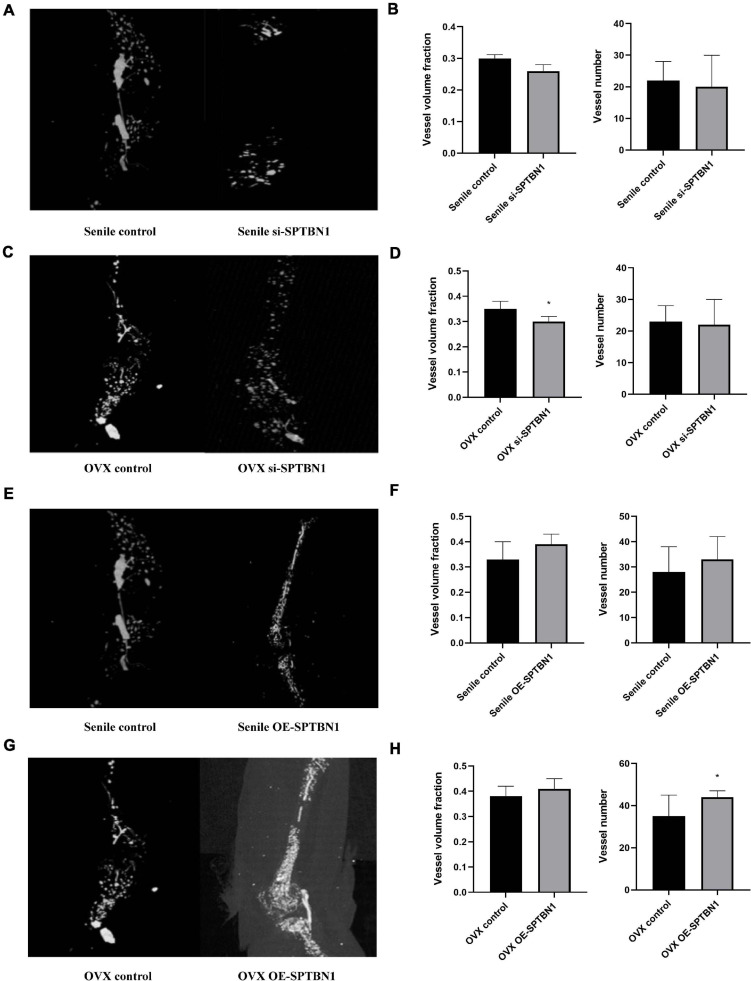
The effect of si-SPTBN1 and OE-SPTBN1 in skeletal blood flow of medullary cavity of primary osteoporosis mice. **(A–C)** Micro-CT scanning of si-SPTBN1 in skeletal blood flow of medullary cavity of senile **(A)** and OVX **(C)** osteoporosis mice. **(B,D)** The vessel volume fraction and vessel numbers were analyzed according to Micro-CT scanning. **(E–H)** Micro-CT scanning of OE-SPTBN1 in skeletal blood flow of medullary cavity of senile **(E)** and OVX **(G)** osteoporosis mice. **(F,G)** The vessel volume fraction and vessel numbers were analyzed according to Micro-CT scanning. Scale bar, 5 μm for all. ^∗^*P* < 0.05, ^∗∗^*P* < 0.01 vs. control group.

Further, we explored the effect of si-SPTBN1 or OE-SPTBN1 in skeletal blood flow during primary osteoporosis. Representative images of three-dimensional characterization of the femur vascularization in the senile and OVX mice with or without si-SPTBN1 injection are shown in Figures 8A,C. The quantitation data indicated that si-SPTBN1 exhibited no significant effect in vessel volume fraction and vessel numbers in the senile mice compared with the control (Figure 8B), and si-SPTBN1 significantly decreased the vessel volume fraction in OVX mice compared with the control group, but with no significant effect in vessel numbers (Figure 8D). The representative images of three-dimensional characterization of the femur vascularization in the senile and OVX mice with or without OE-SPTBN1 injection are shown in Figures 8E,G. The upregulation of SPTBN1 showed no significant effect in vessel volume fraction and vessel numbers in the senile mice compared with the control (Figure 8F), while upregulation of SPTBN1 significantly increased the vessel numbers compared with the control group and showed no significant effect in vessel volume fraction (Figure 8H). Interestingly, although si-SPTBN1 exhibited no significant effect in vessel volume fraction and vessel numbers, there was a significantly decreased trend in the si-SPTBN1 group compared with the control group. Similarly, there was an obvious increasing trend in vessel volume fraction and vessel numbers in the OE-SPTBN1 group compared with the control group.

Vascular endothelial growth factor is a crucial regulator for angiogenesis in developing mature bone tissue (Liu and Olsen, 2014). IHC assay was performed to evaluate the expression of VEGF in the distal femur of mice with different treatments. The representative images of IHC in the senile mice are shown in Supplementary Figure 5A and those of OVX mice are shown in Supplementary Figure 5B. The quantitation data revealed that downregulation of SPTBN1 significantly decreased the VEGF positive cells in both the senile group and OVX group compared with the negative control, while upregulation of SPTBN1 significantly increased the VEGF mean density both in the senile group and OVX group compared with the negative control (Supplementary Figures 5A,B). These results suggested that SPTBN1 inhibited primary osteoporosis by suppressing the skeletal blood flow of bone microvessels and decreasing the expression of VEGF.

## Discussion

The traditional form of treating osteoporosis is by use of drug interventions to inhibit bone resorption or promote bone formation, including bisphosphonates, hormone replacement therapy with estrogen, calcitonin, selective estrogen receptor modulators, denosumab, strontium ranelate, and teriparatide (Baccaro et al., 2015). However, due to the limited therapeutic effects and obvious side effects of these traditional drug interventions, treatment of osteoporosis remains a major clinical challenge. For example, the long-term use of bisphosphonates leads to excessive inhibition of bone remodeling, increased bone fragility, and increased creatinine levels (Alwahhabi and Alsuwaine, 2017). Hormone replacement therapy with estrogen increases the risk of heart disease, venous thrombosis, and breast cancer (Tella and Gallagher, 2014). It is urgent to explore novel safer and targeted active drugs against osteoporosis. However, the development of new molecular diagnostic and therapeutic targets require an increased understanding of the pathogenesis of osteoporosis. Therefore, describing the pathogenesis and underlying mechanisms of osteoporosis and identification of novel drug targets are required in the prevention and treatment of osteoporosis.

Increasing evidences have indicated that bone mass homeostasis starts failing in midlife, resulting in bone loss, osteoporosis, and debilitating fractures (Harada and Rodan, 2003). During this process, osteoblast cells play a crucial role in bone formation and differentiation (Ali et al., 2005). Meanwhile, more and more studies have revealed that an effective strategy to protect against osteoporosis is promoting the proliferation and differentiation of osteoblast (Pan et al., 2018). SPTBN1 is considered to be associated with the progression of osteoporosis (Wang et al., 2012), however, the specific functions and mechanisms remain unclear. In this study, a primary osteoporosis mice model including senile osteoporosis and postmenopausal osteoporosis was established. The expression of SPTBN1 was found to be significantly downregulated both in the senile osteoporosis group and postmenopausal osteoporosis group compared with corresponding control groups. The silencing of SPTBN1 significantly inhibited the proliferation and differentiation and induced the apoptosis of mouse pre-osteoblast MC3T3-E1 cells. However, overexpression of SPTBN1 exhibited an opposite effect of SPTBN1 silencing. These results indicated that SPTBN1 potentially acted as a negative effect of SPTBN1 on osteoporosis progression.

Previous studies have demonstrated that SPTBN1 is a ligand of the Smad3/Smad4 complex in the TGF-β signaling pathway, and SPTBN1 inhibits the TGF-β pathway by downregulating the phosphorylation level of Smad3/Smad4 during the transcription of nucleus regulatory genes (Zhi et al., 2015). Recently, a series of studies found that interfering with the TGF-β signaling pathway can significantly affect the development of osteoporosis. Lin et al. (2019) indicated that acupoint application therapy (AAT) using TianGui Powder (TGP) can prevent the progression of osteoporosis in ovariectomized rats by inhibiting the TGF-β/Smad2/3 signaling pathway. Song et al. (2015) revealed that sulfuretin induces osteoblast differentiation by activating the TGF-β/Smad2/3 pathway. Besides, non-coding RNAs have also been identified to affect bone metabolism and the development of osteoporosis. miR-10b has been demonstrated to stimulate osteogenesis partly by activating the TGF-β/SMAD2 signaling pathway (Li et al., 2018). Jiang et al. (2010) revealed that miR-214-5p promote BMSCs differentiation by modulating the TGF-β/Smad2/COL4A1 pathway, and is a potential target in the development of novel drugs for postmenopausal osteoporosis (Yoshioka and Yoshiko, 2017). In this study, downregulation of SPTBN1 was found to significantly increase the expression of TGF-β and the phosphorylation level of Smad3, while overexpression of SPTBN1 significantly decreased the expression of TGF-β and the phosphorylation level of Smad3. These results suggested that the function of SPTBN1 in the differentiation and function of osteoblasts is mediated by the TGF-β/Smad3 pathway.

As expected for the important roles of TGF-β/Smad3 signaling, the STAT1 molecule played a unique effect in bone metabolism (Wang et al., 2015). Li J. et al. (2015) revealed that miR-184 promoted the differentiation of osteoblasts by downregulating the expression of STAT1. Moreover, Orliæ et al. (2007) found that the expression of STAT1 was significantly upregulated approximately 2.16-fold in the ovariectomized rats when compared with the normal rats. Huang et al. (2016) identified Cxcl9 as an angiostatic factor secreted by osteoblasts in the bone marrow microenvironment, and extended the following mechanisms: Cxcl9 is transcriptionally upregulated by STAT1 in osteoblasts, interacts with vascular endothelial growth factor and prevents its binding to endothelial cells and osteoblasts, thus inhibiting angiogenesis and osteogenesis both *in vivo* and *in vitro*. Our previous study has indicated that the upregulation of STAT1 in osteoblasts can significantly lead to increased secretion of chemokine (C-X-C motif) ligand 9 (Cxcl9), and Cxcl9 can competitively bind to VEGF and block its binding to the receptor on vascular endothelial cells, thus inhibiting the formation of bone vessels (Huang et al., 2016). This reveals the mechanism that osteoblasts affect bone metabolism by regulating bone angiogenesis. Numerous studies have reported the correlation between the STAT1 pathway and the progression of osteoporosis. For instance, interleukin-35 has been reported to suppress TNF-α-induced osteoclastogenesis and induce apoptosis through regulation of the JAK1/STAT1 signaling pathways (Peng et al., 2018). These results confirmed the important function of the STAT1/Cxcl9 pathway in bone metabolism, and its dysregulation can result in the occurrence of osteoporosis. Here, the results showed that the silencing of SPTBN1 significantly increased the expression of Cxcl9 and the phosphorylation level of STAT1, while SPTBN1 overexpression significantly decreased the expression of Cxcl9 and the phosphorylation level of STAT1. Besides, these results suggested that the effect of SPTBN1 in osteoporosis also might be mediated by the STAT1/Cxcl9 signaling pathway.

Previous studies have revealed that bone formation is tightly coupled to angiogenic growth of blood vessels during the development of the mammalian skeletal system (Wang et al., 2007). VEGF, a master regulator of angiogenesis, has been identified to control growth plate morphogenesis, cartilage remodeling, blood vessel invasion and ossification during skeletal development (Maes et al., 2010). In this study, we found that SPTBN1 inhibited primary osteoporosis by suppressing the skeletal blood flow of bone microvessels and decreasing the expression of VEGF. The results extended the specific mechanisms of SPTBN1 in the progression of osteoporosis. However, there are a few limitations to this study: (1) This study was carried out in a primary osteoporosis mouse model and mouse pre-osteoblast MC3T3-E1 cells, and the phenotype and roles of SPTBN1 need to be confirmed under the conditions in patients with osteoporosis; (2) The corresponding inhibitors or antibodies against Smad3 and Cxcl9 should be used to determine the effect of SPTBN1 in the TGF-β/Smad3 and STAT1/Cxcl9 signaling pathways, as well as in the progression of osteoporosis.

## Conclusion

This study specifically extended the regulatory network of SPTBN1 I in primary osteoporosis. The results showed that overexpression of SPTBN1 significantly suppresses the development of primary osteoporosis by modulating VEGF, TGF-β/Smad3, and STAT1/Cxcl9 signaling pathways. This study suggested that SPTBN1 can be considered as a potential biomarker for the diagnosis and treatment of primary osteoporosis.

## Data Availability Statement

The data presented in the study are deposited in the Baidu Netdisk repository, accession number is SPTBN1-database.

## Ethics Statement

The animal study was reviewed and approved by this study was approved by Southern Medical University.

## Author Contributions

XX, JS, JY, and XB: guarantor of integrity of the entire study. JS and XB: study concepts. JS and XX: study design. JY: definition of intellectual content. XW, XC, XF, and GC: literature research. HW: clinical studies. XX, JY, YZ, YY, HW, YS, and GC: experimental studies. XX, JY, YZ, YY, XF, and GC: data acquisition. HW, MF, JY, and GC: data analysis. HW, XL, YS, JY, and GC: statistical analysis. XX and JY: manuscript preparation and manuscript editing. JS and XB: manuscript review. All authors: read and approved the manuscript.

## Conflict of Interest

The authors declare that the research was conducted in the absence of any commercial or financial relationships that could be construed as a potential conflict of interest.

## Supplementary Material

Supplementary Figure 1 The relation among SPTBN1, TGF-β, STAT1/Cxcl9 and VEGF. **(A)** The network among SPTBN1, TGF-β, STAT1/Cxcl9, and VEGF. **(B)** Interactions among SPTBN1, TGF-β, STAT1/Cxcl9, and VEGF in tabular form.

Supplementary Figure 2 The selection of optimal RNAi and overexpressing sequence for SPTBN1 (si-SPTBN1 and OE-SPTBN1). **(A–C)** The selection of optimal RNAi sequence for SPTBN1. Primary osteoblast MC3T3-E1 cells were transfected with different RNAi sequences for SPTBN1 including 200, 201, and 202. **(A)** The transfection fluorescence rate after 48 h of transfection observed using a fluorescence microscope. **(B)** The mRNA expression of SPTBN1 after 72 h of transfection evaluated by qRT-PCR (*N* = 3). **(C)** The protein expression of SPTBN1 after 2 h of transfection evaluated by Western blot analysis (*n* = 3). **(D–F)** The selection of optimal overexpressing sequence for SPTBN1. Primary osteoblast MC3T3 cells were transfected with different overexpressing sequences for SPTBN1 including 6164, 6165, and 6166. **(D)** The transfection fluorescence rate after 48 h of transfection observed using a fluorescence microscope. **(E)** The mRNA expression of SPTBN1 after 72 h of transfection evaluated by qRT-PCR (*N* = 3). **(F)** The protein expression of SPTBN1 after 2 h of transfection evaluated by Western blot analysis (*n* = 3). ^∗^*P* < 0.05, ^∗∗^*P* < 0.01.

Supplementary Figure 3 The effect of si-SPTBN1 and OE-SPTBN1 in osteoblasts proliferation in the distal femur of primary osteoporosis mice. **(A)** ALP staining of the distal femur of si-SPTBN1 or OE-SPTBN1 transfected senile osteoporosis mice. **(B)** ALP staining of the distal femur of si-SPTBN1 or OE-SPTBN1 transfected OVX osteoporosis mice. **(C)** Quantification of ALP positive cells per bone area according to **(A)**. **(D)** Quantification of ALP positive cells per bone area according to **(B)**. Arrows indicate the ALP positive cells. Scale bar, 5 μm for all. ^∗^*P* < 0.05, ^∗∗^*P* < 0.01.

Supplementary Figure 4 The effect of si-SPTBN1 and OE-SPTBN1 in osteoclasts proliferation in the distal femur of primary osteoporosis mice. **(A,B)** TRAP staining of the distal femur of si-SPTBN1 **(A)** and OE-SPTBN1 **(B)** transfected senile osteoporosis mice. Quantification of TRAP-positive cells per bone area. **(C,D)** TRAP staining of the distal femur of si-SPTBN1 **(C)** and OE-SPTBN1 **(D)** transfected OVX osteoporosis mice. Quantification of TRAP-positive cells per bone area. Scale bar, 20 μm for all. ^∗^*P* < 0.05, ^∗∗^*P* < 0.01.

Supplementary Figure 5 The effect of si-SPTBN1 and OE-SPTBN1 in the expression of VEGF in distal femur of primary osteoporosis mice. Immunohistochemistry staining for VEGF in the distal femur of si-SPTBN1 or OE-SPTBN1 transfected senile **(A)** and OVX **(B)** osteoporosis mice. The mean density of the respective positive cells was calculated as integrated optical density (IOD) per area of positive cells. The magnification of photomicrographs of immunohistochemistry was 40×. Arrows indicate the VEGF positive cells stained in brown. Scale bar, 5 μm for all. *N* = 8. ^∗^*P* < 0.05, ^∗∗^*P* < 0.01.
