# Supplementary figures and images for: SPTBN1 Prevents Primary Osteoporosis by Modulating Osteoblasts Proliferation and Differentiation and Blood Vessels Formation in Bone
Source: Front Cell Dev Biol. 2021 Mar 19;9:653724. doi: 10.3389/fcell.2021.653724 (PMC8017174; doi:10.3389/fcell.2021.653724)

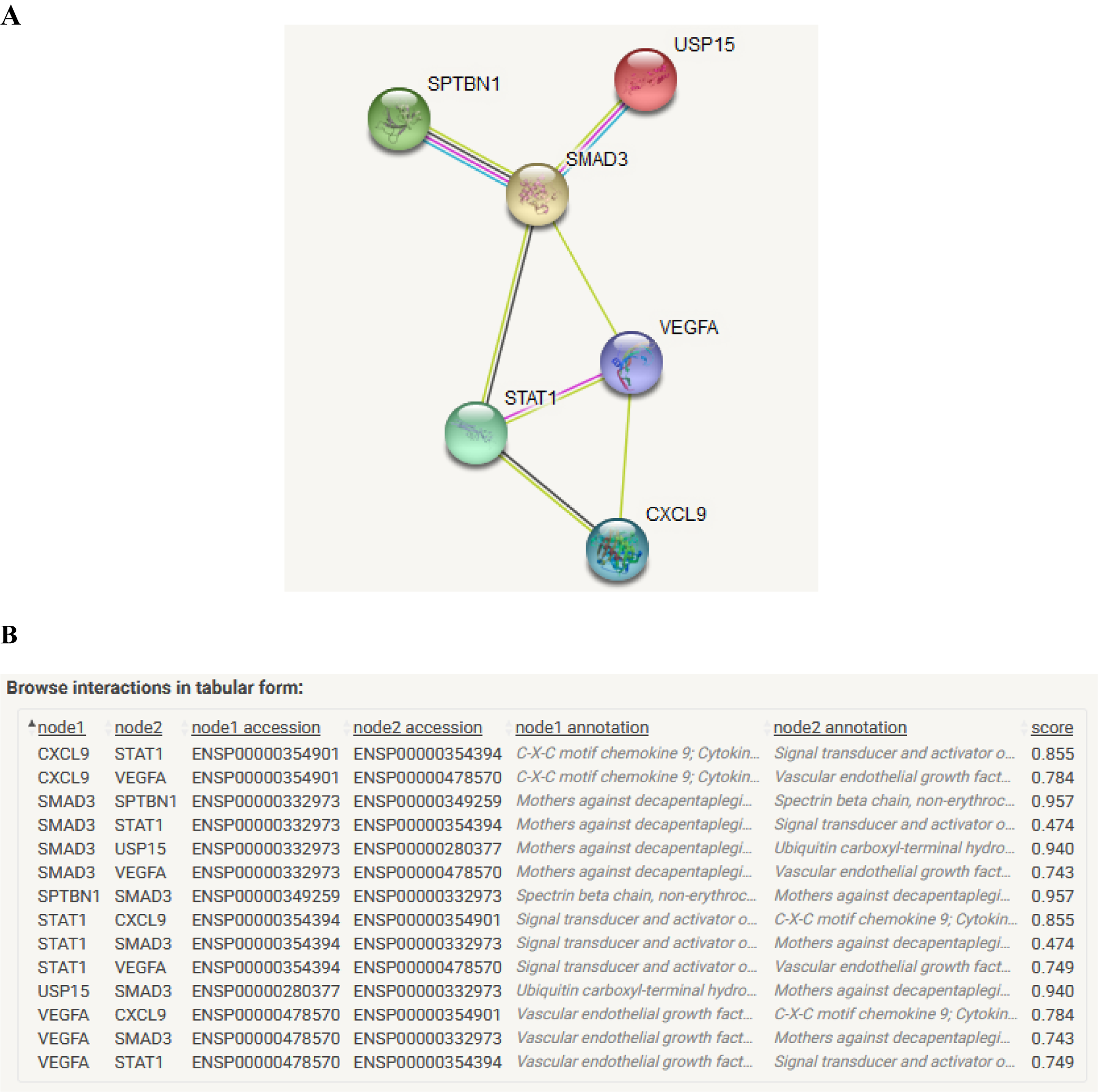

Supplement: Supplementary Figure 1 — The relation among SPTBN1, TGF-β, STAT1/Cxcl9 and VEGF. (A) The network among SPTBN1, TGF-β, STAT1/Cxcl9, and VEGF. (B) Interactions among SPTBN1, TGF-β, STAT1/Cxcl9, and VEGF in tabular form. [file Image_1.TIF]

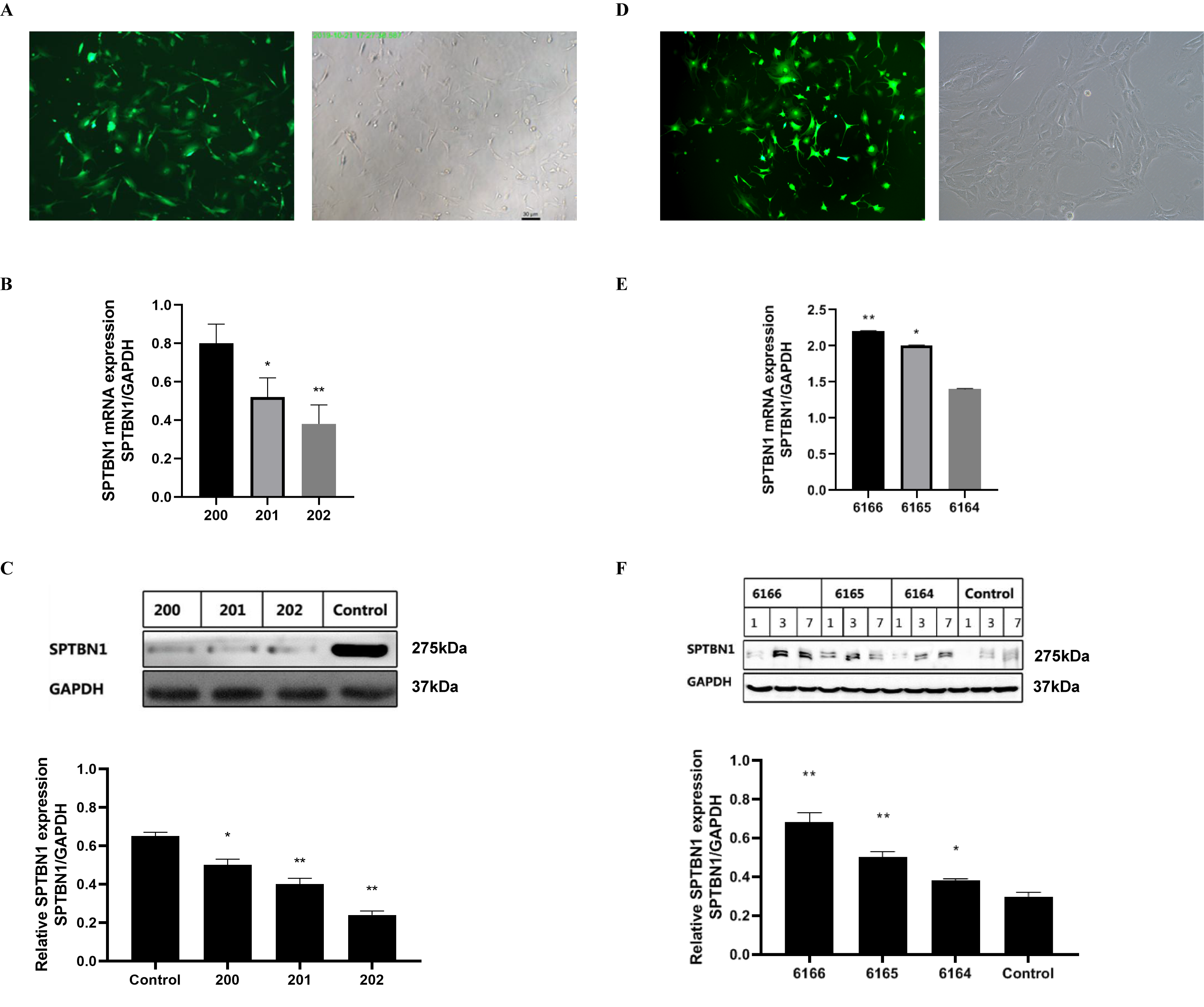

Supplement: Supplementary Figure 2 — The selection of optimal RNAi and overexpressing sequence for SPTBN1 (si-SPTBN1 and OE-SPTBN1). (A–C) The selection of optimal RNAi sequence for SPTBN1. Primary osteoblast MC3T3-E1 cells were transfected with different RNAi sequences for SPTBN1 including 200, 201, and 202. (A) The transfection fluorescence rate after 48 h of transfection observed using a fluorescence microscope. (B) The mRNA expression of SPTBN1 after 72 h of transfection evaluated by qRT-PCR (N = 3). (C) The protein expression of SPTBN1 after 2 h of transfection evaluated by Western blot analysis (n = 3). (D–F) The selection of optimal overexpressing sequence for SPTBN1. Primary osteoblast MC3T3 cells were transfected with different overexpressing sequences for SPTBN1 including 6164, 6165, and 6166. (D) The transfection fluorescence rate after 48 h of transfection observed using a fluorescence microscope. (E) The mRNA expression of SPTBN1 after 72 h of transfection evaluated by qRT-PCR (N = 3). (F) The protein expression of SPTBN1 after 2 h of transfection evaluated by Western blot analysis (n = 3). ∗P < 0.05, ∗∗P < 0.01. [file Image_2.TIF]

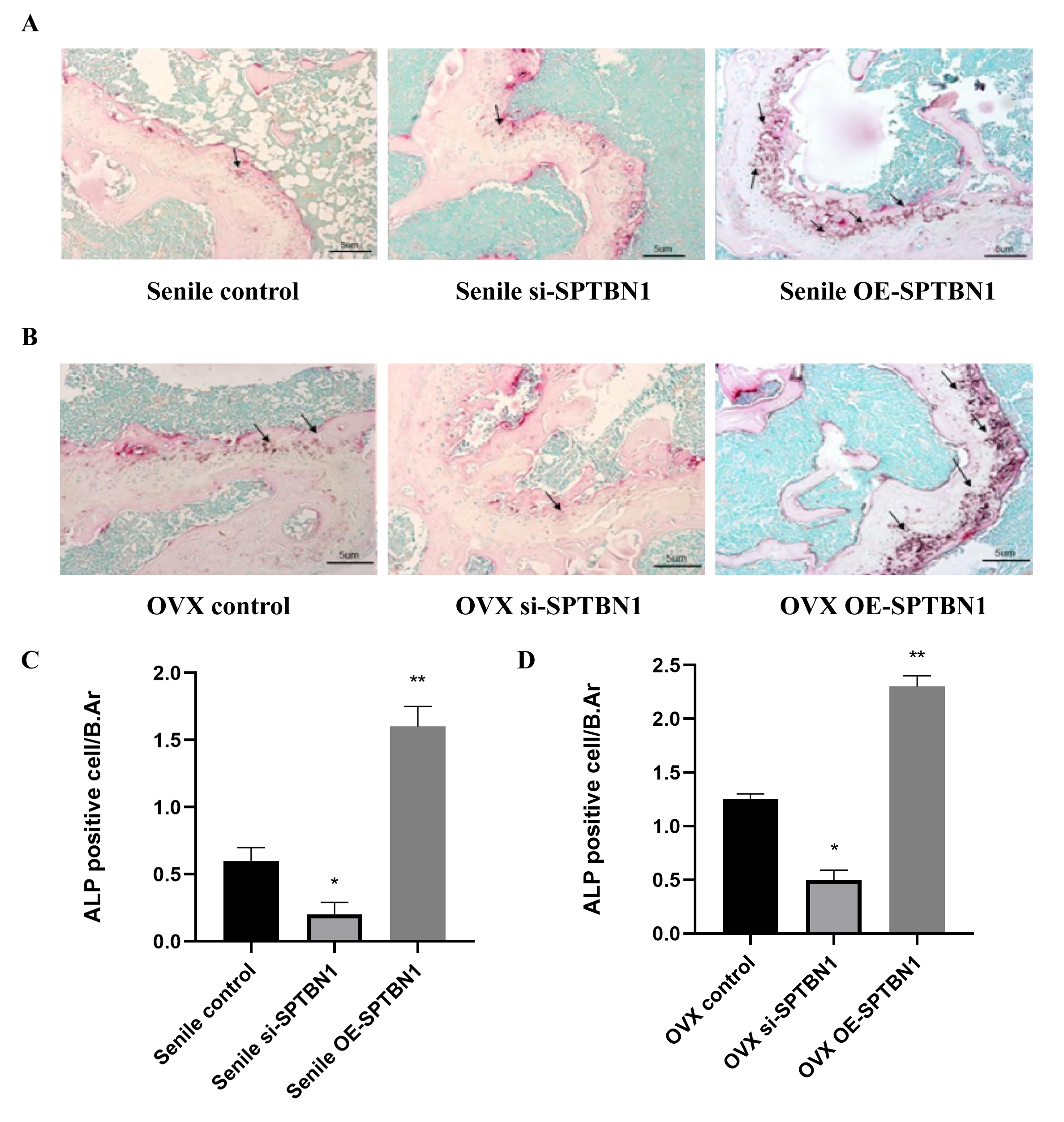

Supplement: Supplementary Figure 3 — The effect of si-SPTBN1 and OE-SPTBN1 in osteoblasts proliferation in the distal femur of primary osteoporosis mice. (A) ALP staining of the distal femur of si-SPTBN1 or OE-SPTBN1 transfected senile osteoporosis mice. (B) ALP staining of the distal femur of si-SPTBN1 or OE-SPTBN1 transfected OVX osteoporosis mice. (C) Quantification of ALP positive cells per bone area according to (A). (D) Quantification of ALP positive cells per bone area according to (B). Arrows indicate the ALP positive cells. Scale bar, 5 μm for all. ∗P < 0.05, ∗∗P < 0.01. [file Image_3.TIF]

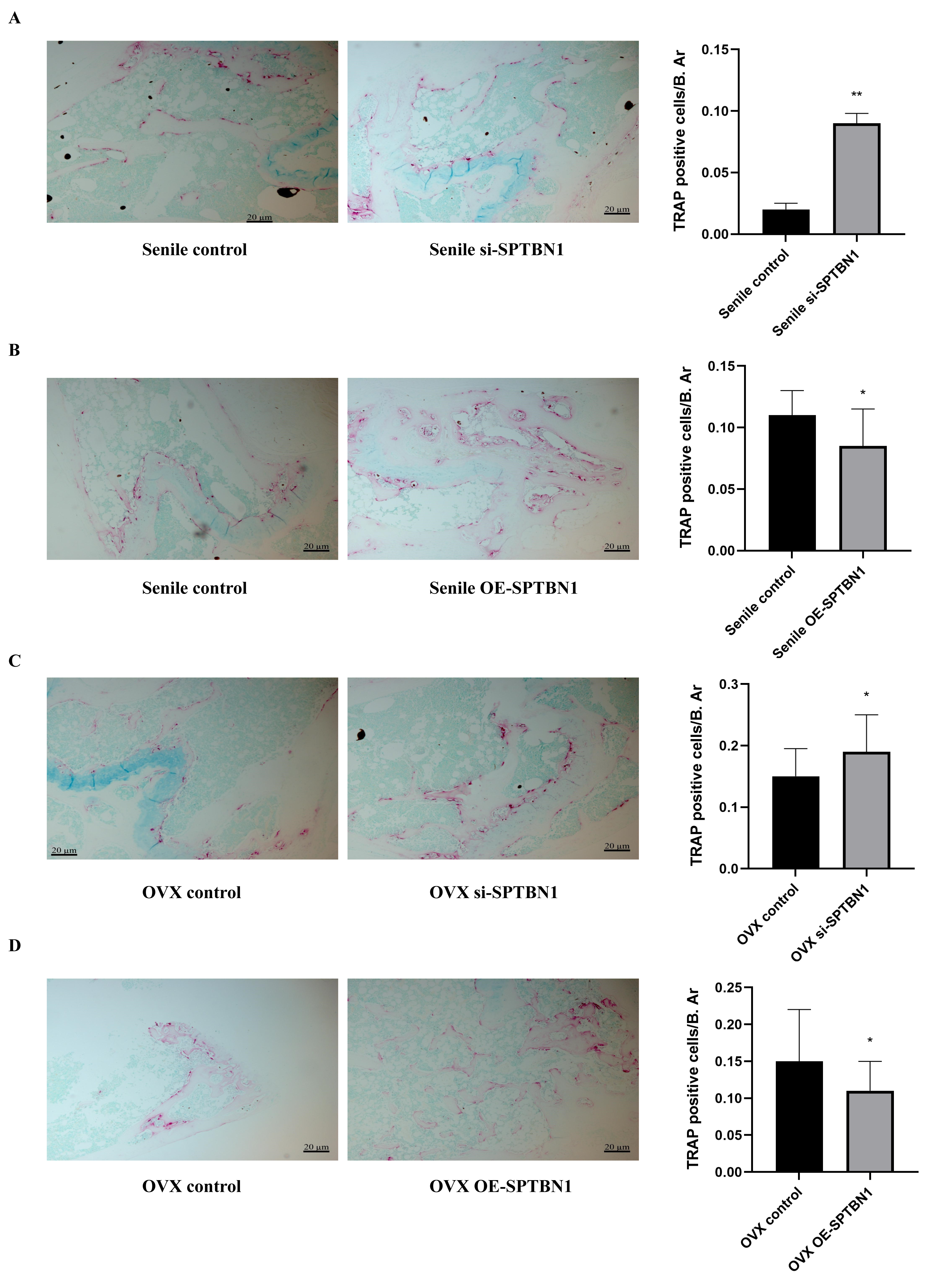

Supplement: Supplementary Figure 4 — The effect of si-SPTBN1 and OE-SPTBN1 in osteoclasts proliferation in the distal femur of primary osteoporosis mice. (A,B) TRAP staining of the distal femur of si-SPTBN1 (A) and OE-SPTBN1 (B) transfected senile osteoporosis mice. Quantification of TRAP-positive cells per bone area. (C,D) TRAP staining of the distal femur of si-SPTBN1 (C) and OE-SPTBN1 (D) transfected OVX osteoporosis mice. Quantification of TRAP-positive cells per bone area. Scale bar, 20 μm for all. ∗P < 0.05, ∗∗P < 0.01. [file Image_4.TIF]

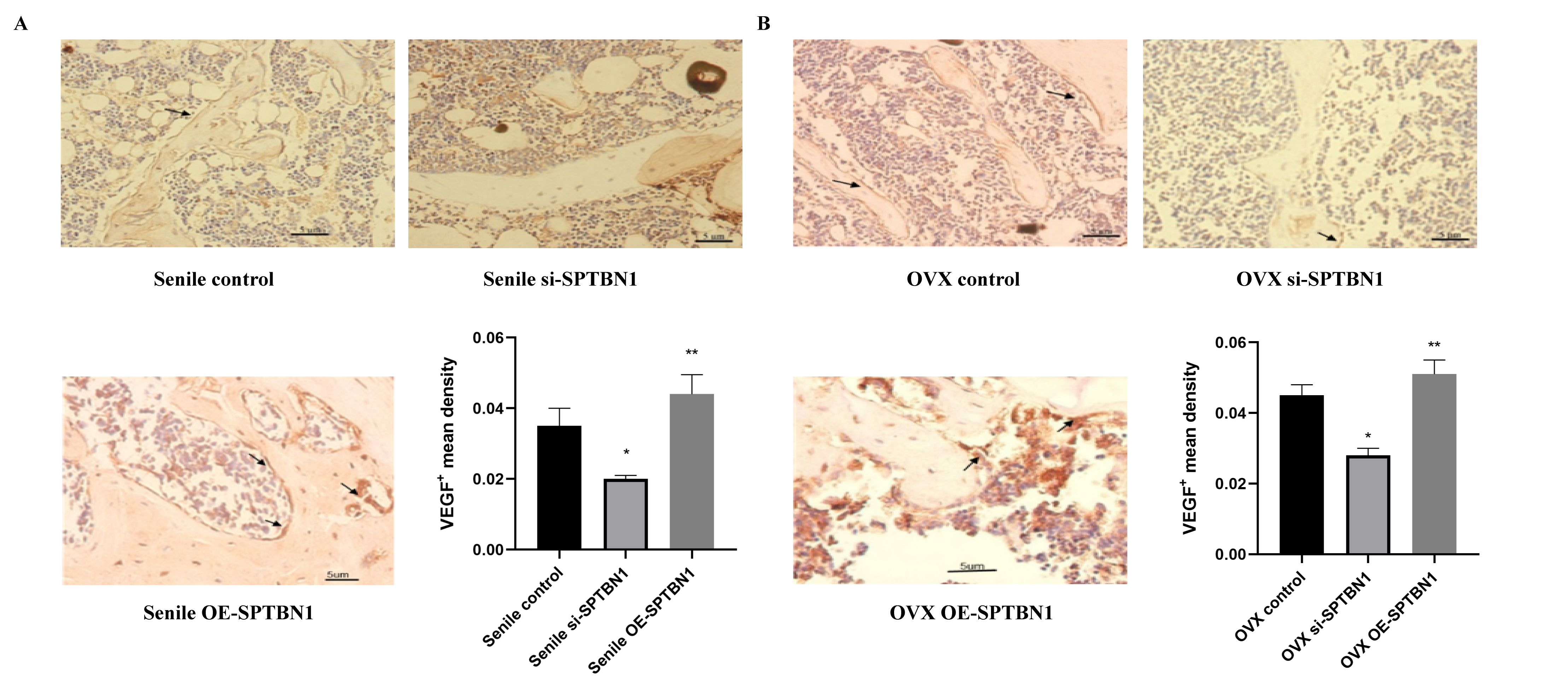

Supplement: Supplementary Figure 5 — The effect of si-SPTBN1 and OE-SPTBN1 in the expression of VEGF in distal femur of primary osteoporosis mice. Immunohistochemistry staining for VEGF in the distal femur of si-SPTBN1 or OE-SPTBN1 transfected senile (A) and OVX (B) osteoporosis mice. The mean density of the respective positive cells was calculated as integrated optical density (IOD) per area of positive cells. The magnification of photomicrographs of immunohistochemistry was 40×. Arrows indicate the VEGF positive cells stained in brown. Scale bar, 5 μm for all. N = 8. ∗P < 0.05, ∗∗P < 0.01. [file Image_5.TIF]
